# Editorial: The emerging role of liquid biopsy in gastrointestinal, pancreatic and liver cancers

**DOI:** 10.3389/fmed.2023.1341739

**Published:** 2023-12-18

**Authors:** Razvan Iacob, Doru Paul, Irinel Popescu

**Affiliations:** ^1^“Carol Davila” University of Medicine and Pharmacy, Bucharest, Romania; ^2^Digestive Diseases and Liver Transplantation Center, Fundeni Clinical Institute, Bucharest, Romania; ^3^Center for Excellence in Translation Medicine, Fundeni Clinical Institute, Bucharest, Romania; ^4^Division of Hematology and Medical Oncology, Department of Medicine, Weill Cornell Medicine/New York-Presbyterian, New York, NY, United States

**Keywords:** liquid biopsy, gastrointestinal cancer, liver cancer, pancreatic cancer, prognosis, early diagnosis, personalized therapy

Liquid biopsy (LB) has emerged as a promising tool in oncology, assisting clinicians in early cancer detection, treatment selection, and monitoring of tumor progression. LB provides a non-invasive, dynamic picture of the cancer progression and tumor burden (1). However, technological challenges and high costs make its wide clinical adoption still challenging (2). Recent data regarding clinical utility of circulating tumor cells (CTCs) in diagnosis and prognosis of patients with gastrointestinal cancers, as predictors of progression-free survival or poor outcome has been published (3). Also, ctDNA, derived from various biological processes like cancer cells apoptosis or micro-metastasis, offers insights into genetic and epigenetic cancer alterations. It is a source of valuable information on tumor genomics, distinguishing tumor molecular subtypes, and guiding therapy (4). The analysis of ncRNAs, including miRNAs and lncRNAs, could also, potentially, have diagnostic, prognostic, and therapeutic utility. These molecules, often secreted in the bloodstream and other body fluids, reflect the primary tumor's expression profile and are associated with disease grading and malignant progression (5).

Colorectal cancer is the first solid tumor where the concept of minimal residual disease (MRD) lead to a change in clinical practice (6). Since 2022, ctDNA testing after surgery has been approved by Medicare, the main insurer in the United States. The benefit of LB in informing colorectal cancer management has been reviewed recently (7–9) and, therefore, the prime focus of the five articles included in this Research Topic is to present the utility of LB (LB) in several non-colorectal gastrointestinal (GI) malignancies.

First, Iacob et al. provide a systematic review of LB biomarkers for esophageal squamous cell carcinoma (ESCC) alongside with head and neck squamous cell carcinoma (HNSCC) as both cancers share common risk factors and embryological origins, while having distinct prognoses. LB research could significantly aid in finding early diagnosis biomarkers in these diseases, as both HNSCC and ESCC are often detected at advanced stages, leading to high morbidity and low survival rates. No current clinical guideline recommends biomarkers for early detection of either one of these cancers in patients at-risk. This review is the first one, to our knowledge, that explores the utility of LB in two closely related cancers, focusing on shared molecular markers. While CTCs have a documented prognostic utility, the recently FDA-approved Cellsearch platform appears promising for clinical integration of CTCs detection and quantification for both ESCC and HNSCC. CtDNA analysis, including methylation profiles and TP53 somatic alterations, might be beneficial for early diagnosis. MiRNAs also may be useful for early diagnosis and prognosis both in ESCC and in HNSCC, due to MiRNAs function as regulators of cell proliferation. In particular, MiR-10, that targets TIAM, is a promising biomarker, with good sensitivity and specificity, and in the future, it may be introduced in the clinic for both ESCC and HNSCC diagnosis. Further research is needed in this area as currently no single biomarker can robustly differentiate between ESCC and HNSCC and be recommended routinely for screening, early detection, and prognostic evaluation.

Gastric cancer (GC) is the fifth most common cancer worldwide, with very high morbidity and mortality rates. Traditional serum biomarkers for GC, such as CEA, CA19-9, CA-125, and CA72-4, have low sensitivity and only modest specificity, limiting their diagnostic effectiveness. Chivu-Economescu et al. provide, in their review, an in-depth analysis of the advancements in LB technology for GC diagnosis and management. Several LB biomarkers, like CTCs, especially if associated with HER2, or PD-L1 amplification or positivity, respectively, also ctDNA alterations and, more recently, exosomes and their cargo, have demonstrated clinical utility, supporting their potential role in early diagnosis, personalized therapy and prognostic estimation.

In their review, Osei-Bordom et al. have focused on the clinical utility of liquid biopsies in pancreatic ductal adenocarcinoma (PDAC), one of the cancers with the highest worldwide mortality. Due to its late presentation, PDAC is one of the most challenging human malignancies, as it leads to extremely low survival rates. The paper explores how LB, including CTCs, circulating free nucleic acids (cfDNA, cfRNA, cfmiRNA), exosomes, and tumor-educated platelets (TEPs) add valuable insights into the early detection and monitoring of PDAC. As it is often diagnosed in late stages of the disease, when availability of tissue biopsy becomes problematic, LB could be of particular interest for PDAC. LB allows access to PDAC's genomic profile, confirming its heterogeneity and assisting in early detection, surveillance, treatment monitoring, and prognostic estimation. The RNA profiles of TEPs are altered in the presence of cancer, differentiating PDAC from healthy tissue. Similarly, circulating tumor RNA (ctRNA) is investigated for its role in expressing specific non-coding regions of PDAC tumors' genetic transcripts.

Manea et al. focus on LB as a promising tool for management of hepatocellular carcinoma (HCC). Plasma LB, in particular, offers advantages over traditional serum biomarkers like alpha-fetoprotein (AFP) for HCC screening, monitoring tumor dynamics, and therapeutic response. CfDNA in liquid biopsies can identify HCC through unique methylation patterns, somatic mutations, and fragmentome characteristics. However, challenges include the need for standardized cfDNA extraction and analysis, difficulty in detecting low-frequency cfDNA features, and the complexity of interpreting molecular data. Extracellular vesicles (EVs), such as exosomes, also hold potential for early HCC detection, carrying biomarkers like miRNAs and lncRNAs. EVs protection offer stability for these biomarkers in circulation but isolating tumor-specific EVs from background non-tumor EVs remains demanding, requiring further standardization. Combining EV-based biomarkers with other LB methods or traditional serology could enhance diagnostic accuracy for HCC. The future of LB in HCC diagnosis depends on refining isolation and analysis methods, reducing costs, and validating clinical utility in large-scale studies. The optimal LB component or combination for early HCC detection is yet to be determined.

The integration of blood LB in GI cancer management, with an emphasis on clonal hematopoiesis (CH) as a potential source of biological noise, is the main objective of the paper of Croitoru et al.. CH, resulting from the accumulation of somatic mutations and clonal proliferation of hematopoietic stem cells with aging, can lead to misinterpretation of cancer-related mutations in LB, impacting diagnosis and treatment (Croitoru et al.). The presence of non-cancer mutations in cfDNA, including CH mutations, poses challenges in the analysis of genomic data. CH mutations, affecting genes like KRAS, PIK3CA, and EGFR, can be misclassified as tumor-related, leading to incorrect patient management. The review outlines the clinical importance of CH in GI cancer patients and the need for careful interpretation of LB results, to distinguish between tumor-derived ctDNA and other genetic alterations, such as CH mutations. It underscores the significance of correctly identifying CH mutations to prevent mismanagement in GI cancer patients and the need for better understanding and standardization in LB analysis.

In summary, the use of LB in gastrointestinal oncology is evolving very fast, and, as technological breakthroughs and novel bioinformatic solutions become widely available, they will soon routine clinical practice, leading to more precise cancer diagnosis, informing personalized treatments and more accurate prognostic estimation.

## Author contributions

RI: Conceptualization, Writing – original draft, Writing – review & editing. DP: Supervision, Validation, Writing – review & editing. IP: Supervision, Validation, Writing – review & editing.
